# Case report: Surgical resection of a retro-hepatic leiomyosarcoma involving atrial reconstruction, cardiopulmonary bypass, *ex vivo* tumor resection, and liver re-implantation

**DOI:** 10.3389/fsurg.2022.1037312

**Published:** 2022-11-07

**Authors:** Neel K. Sharma, Uchenna Okakpu, Jeevan Murthy, Lawrence M. Wei, Roberto Lopez-Solis, Carl Schmidt, Vinay Badhwar, J. Wallis Marsh

**Affiliations:** ^1^ Department of Surgery, West Virginia University, Morgantown, WV, United States; ^2^ School of Medicine, West Virginia University, Morgantown, WV, United States; ^3^ Cardiovascular and Thoracic Surgery, West Virginia University, Morgantown, WV, United States

**Keywords:** *ex vivo* repair, en-bloc resection, inferior vena cava, leiomyosarcoma, retroperitoneum, right atrial anastomosis, hepatic vein anastomosis

## Abstract

**Introduction:**

Leiomyosarcomas (LMS) involving the inferior vena cava (IVC) is a clinically rare entity, accounting for approximately 0.5% of all adult sarcomas.

**Case presentation:**

A 67-year-old male presented to the emergency department with mild back and lower abdominal pain. During the workup, a computed tomography scan without contrast showed an area of decreased attenuation within the liver adjacent to the intrahepatic IVC. Magnetic resonance imaging confirmed the involvement of the retro-hepatic IVC; biopsy confirmed the diagnosis of LMS. Given the location of the involvement of the retro-hepatic IVC, liver explantation was deemed necessary for adequate tumor resection. The superior extension of the tumor toward the heart necessitated Cardio-Pulmonary (CPB). The patient successfully underwent a complex surgical procedure involving liver explantation with *ex vivo* back-table resection of the retro-hepatic LMS, replacement of the retro-hepatic vena cava with a ringed Gore-Tex graft, liver re-implantation, and hepatic vein-atrial reconstruction under cardiopulmonary bypass. There were no intraoperative or post-op complications.

**Discussion:**

The role of vascular reconstruction of the IVC varies depending on the level and extent of the tumor, with options ranging from primary repair, ligation, or reconstruction dictated. Surgical resection with negative margins remains the treatment of choice due to the lack of efficacy of adjuvant therapies. Importantly, liver explantation offers a chance for complete surgical resection and reconstruction. Similarly, the complex nature of the tumor necessitated a pioneering approach involving direct hepato-atrial venous anastomosis.

**Conclusion:**

To the best of our knowledge, this is the first reported case in which the hepatic veins were anastomosed directly to the right atrium while also replacing the native vena cava with a separate graft.

## Introduction

Retro-hepatic inferior vena cava (IVC) leiomyosarcoma is a rare condition with special anatomic considerations that make resection and reconstruction difficult. Case reports detailing successful tumor removal are few (in the hundreds), speaking to the condition’s rarity and the expertise required to perform a successful resection (1, 2). The techniques of resection described in the literature include:

• En-bloc liver and IVC resection.
• Resection of the retro-hepatic IVC with or without reconstruction.
• Chemoradiation therapy.

Of these, the most efficacious treatment is surgical resection, which confers a long-term survival benefit (3). We describe herein an *ex vivo*, retro-hepatic vena cava excision with liver re-implantation and reconstruction of the IVC, including a novel method of hepatic vein grafts to an atrial anastomosis.

## Case description

### Pre-operative patient evaluation

**Figure 1:**
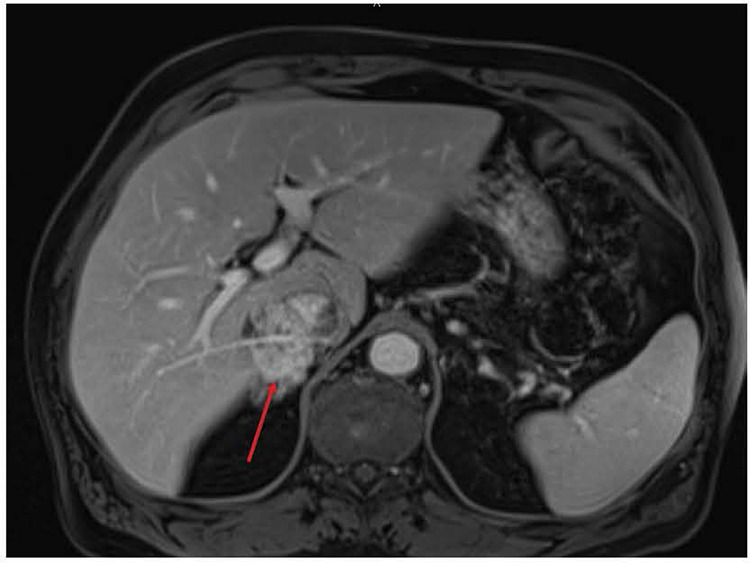
Pre-operative MRI showing involvement of the retro-hepatic IVC by leiomyosarcoma (red arrow). IVC, inferior vena cava.

A 67-year-old male patient presented to his local emergency department for evaluation of lower abdominal pain. He was prescribed antibiotics with minimal relief. Upon return for re-evaluation, a CT scan found him to have a large retro-hepatic mass involving the IVC. An abdomen MRI then uncovered findings concerning sarcoma of the retro-hepatic IVC (Figure 1). A biopsy confirmed the diagnosis of leiomyosarcoma.

The patient was referred to our surgical oncology clinic, where he received a physical exam, which resulted in unremarkable showings; he denied any lower extremity swelling and was found without signs or symptoms of IVC compression. The consulted cardiac surgeons obtained a negative cardiac catheterization. A CT of the chest did not show metastatic disease. The clinic offered total liver explantation with back-table resection of the retro-hepatic IVC and tumor with a subsequent liver re-implantation on cardiopulmonary bypass.

### Intraoperative management

The surgeons performed a sternal incision, isolated the intrapericardial IVC, and then extended the incision to below the umbilicus. After liver mobilization and gallbladder removal, the common duct, cystic artery and duct were ligated and divided. Dissection of the hepatic artery followed in the operation and moved to ligation and division of the gastroduodenal artery. The portal vein was then dissected in preparation for veno-venous bypass. For explantation, the IVC was mobilized completely out of the retroperitoneum.

**Figure 2:**
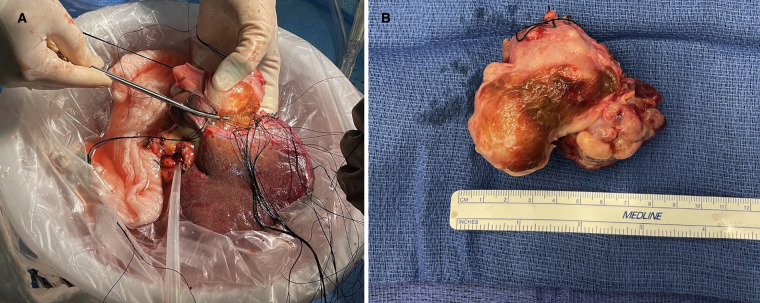
(**A**) Shows explanted liver on the back-table where the inferior IVC and leiomyosarcoma are being resected. (**B**) Shows the resected leiomyosarcoma and IVC. IVC, inferior vena cava.

The patient was then placed on full cardiopulmonary bypass to excise the liver and vena cava. Each was excised from above the renal veins to below the atrium, before being taken to the back-table for tumor resection. At that point, the patient was placed on portal venous bypass, which was connected to the cardiopulmonary bypass circuit. The leiomyosarcoma was circumferentially dissected en-bloc with the IVC, leaving only the origins of the right hepatic vein and the confluence of the middle and left hepatic veins (Figures 2A,B). The cardiac surgeons anastomosed a 20 mm ringed Gore-Tex (GTX) interposition graft from above the renal veins to the IVC, below the diaphragm. They completed the *in situ* native vena reconstruction during back-table work.

After completing the IVC tumor resection, two separate, non-ringed 20 mm GTX grafts were utilized to reconstruct the hepatic veins before re-implantation. One graft was sewn to the origin of the right hepatic vein, and a separate graft was sewn to the confluence of the middle and left hepatic veins. These two grafts were then joined together to allow a single anastomosis directly to the right atrium anterior to the previously placed vena cava ringed GTX graft. Next, the surgeons removed the patient from the portal venous bypass. They re-anastomosed the portal vein end-to-end and perfused the liver thereafter. The patient was able to be weaned from cardiopulmonary bypass at this time.

The procedure moved to hepatic artery anastomosis in another end-to-end fashion at the level of the gastroduodenal artery. Protamine was administered, and the patient was decannulated. After achieving meticulous hemostasis, the biliary anastomosis was performed end-to-end over a biliary stent. A segment of the patient’s pericardium was harvested and utilized to patch the diaphragmatic opening triangularly, preventing compression of the GTX venous grafts. Last, the surgeons placed bilateral chest tubes along with an abdominal Blake drain in the patient prior to closing the pericardium, chest, and abdomen. The transesophageal echocardiogram revealed normal biventricular function. A total of one unit of packed red blood cells and one unit of cryoprecipitate were administered throughout the procedure.

### Post-operative care

**Figure 3:**
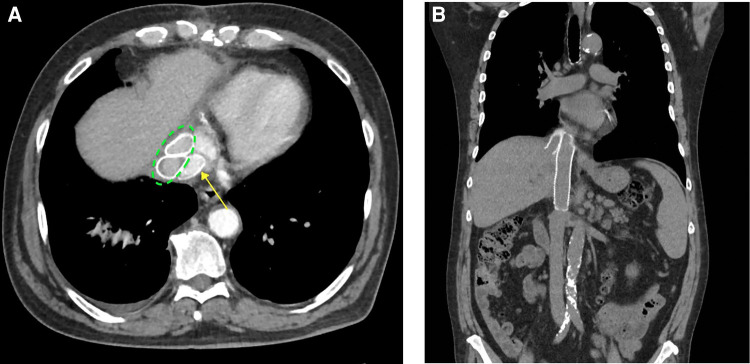
(**A**) Axial images showing the native vena cava GTX graft (posterior) (yellow arrow) and the two anterior hepatic vein GTX grafts (dotted green ellipse). (**B**) Coronal images showing the native vena cava graft (medial) and one of the hepatic vein GTX grafts (lateral). GTX, Gore-Tex.

The patient was extubated on the first post-operative day. His chest tubes were removed on the third post-operative day, and the intra-abdominal drain was removed on day 4. He was discharged home in stable condition on day 5. Other than intractable singultus, the hospital stay was usual. The patient’s 3-month follow-up visit was unremarkable, as well as his 3-month post-operative abdominal and chest CT scans (Figures 3A,B).

## Discussion

Leiomyosarcomas (LMS) are classified as malignant neoplasms involving mesenchymal smooth muscle cells found within the vasculature, gastrointestinal tract, and portions of the genitourinary system. LMS originating from the IVC is extremely rare, representing 0.5% of all diagnosed sarcomas (estimated incidence of 1/100,000) (4). According to Roland et al., 5-year disease-specific survival rates after tumor resection were 65% (5). A retrospective chart review by Teixeira et al. compiled a range of 31%–66.7% for post-resection cava leiomyosarcoma patients (6).

The most common presentation of retro-hepatic cava leiomyosarcoma is abdominal pain; other regularly presenting symptoms are non-specific and include weight loss and fatigue. Site-specific presentation is typically due to venous outflow obstruction (7). Leiomyosarcoma of the IVC is stratified by location into segments I–III:

• Segment I involves the IVC inferior to the renal veins; lesions can cause nephrotic syndrome due to compression or occlusion of the renal vein(s).
• Segment II involves the IVC from the renal veins to the hepatic veins; lesions are typically asymptomatic and are the most common.
• Segment III involves the IVC superior to the hepatic veins. Segment III leiomyosarcoma can present with cardiac arrhythmia and Budd-Chiari syndrome (8, 9). (In all cases, there is an additional risk of lower extremity edema).

The main treatment of IVC leiomyosarcoma is surgical; however, resection and reconstruction techniques vary and are dependent on several factors (10). Surgical techniques include excision of the IVC, explantation of the liver with resection of the cava, resection of the cava utilizing total vascular exclusion with hypothermia, and liver transplantation with or without reconstruction of the IVC (2, 8, 11, 12). If resection is performed, reconstruction of the IVC may or may not be necessary, depending on the degree and duration of obstruction and the presence of venous collaterals (2, 13).

**Figure 4:**
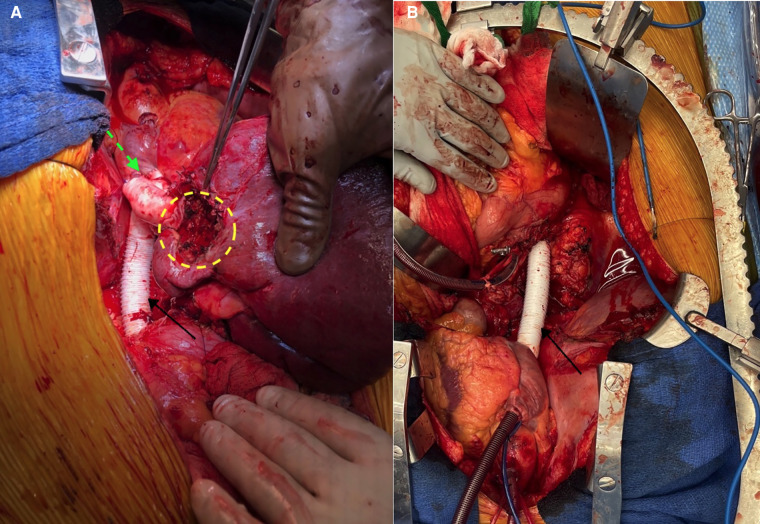
(**A**) Intraoperative view of the various graft reconstructions. Dotted green arrow = site of hepato-atrial anastomosis; dotted yellow circle = region of excised tumor; solid black arrow = vena caval GTX graft. (**B**) Vena caval graft (solid black arrow) before liver re-implantation. GTX, Gore-Tex.

In our case, the lack of radiographically significant local invasion of the patient’s segment II tumor made the *ex vivo* approach to expose and resect the lesion a viable option. This option facilitated an end-to-end vena cava prosthetic graft anastomosis. Furthermore, the hepatic veins were amenable to anastomosis directly to the atrium. Through our literature review, this hepatic vein GTX conduit to atrium anastomosis appears to be a novel method of reconstruction not previously described. This pioneering approach to anastomosis was necessary for practical reasons. Because of the involvement of the hepatic veins by the leiomyosarcoma, the confluence of the hepatic veins had to be resected and reconstructed with two GTX grafts (one to the right and one to the confluence of the middle and left). The native vena cava between the renal veins and the atrium also required resection; this was subsequently reconstructed with a GTX graft in the heterotopic position. To reimplant the joined hepatic vein grafts into the vena, the caval graft would have required lengthy extensions to the hepatic vein grafts. To reach the vena caval grafts, the hepatic vein grafts would have to have been angled significantly posteriorly and would have been in danger of kinking with subsequent thrombosis. The atrial anastomosis was a straight shot for this anastomosis which allowed a much shorter length of hepatic vein GTX grafts and eliminated the risk of kinking/thrombosis. Intraoperative images shown in Figure 4 highlight further details of the reconstruction.

Chemotherapy, which was previously thought to be of limited benefit in the treatment of cava leiomyosarcoma, has now been shown to have increased efficacy in the modern area of chemotherapeutics. Squires et al. performed a single-center experience evaluating neoadjuvant and adjuvant chemotherapy in patients diagnosed with cava leiomyosarcoma. They found that out of 11 patients evaluated, two were able to convert from an unresectable disease to an R1 resection with the addition of neoadjuvant tyrosine kinase inhibitor-based chemotherapy. Additionally, through proteomic analysis, *MYH11* also emerged as a potential diagnostic marker (14). Hines et al. noticed that in a single-center review when patients were treated with multimodality therapy (radical resection, radiation, or chemotherapy), the 10-year survival of IVC leiomyosarcoma was greater than leiomyosarcoma of the stomach, small intestine, retroperitoneum, or uterus (15). The role of radiation therapy and chemotherapy is difficult to fully characterize due to the rarity of the disease and the lack of ability to perform a randomized control trial.

The patient remains grateful for the procedure and continues to do well following surgery. Recently, he started showing radiological signs of metastatic disease following the discovery of a lung mass, but appreciates that the surgical resection and reconstruction have not only enhanced his quality of life, but have also potentially helped increase his overall survival. In his words, “The surgical team helped me enjoy a memorable Christmas with my family, that is what it’s all about!”

## Conclusion

The challenging anatomic considerations involved in treating IVC LMS have led to numerous techniques for resection and reconstruction. However, no significant difference in disease-free survival has been noted based on excision or reconstruction techniques, allowing the surgeon to tailor the treatment with considerations for disease progression, local invasion, and surgeon expertise.

## Data Availability

The original contributions presented in the study are included in the article/Supplementary Material, further inquiries can be directed to the corresponding author/s.
